# Supplementary material for: Hot spot prediction in protein-protein interactions by an ensemble system
Source: BMC Syst Biol. 2018 Dec 31;12(Suppl 9):132. doi: 10.1186/s12918-018-0665-8 (PMC6311905; doi:10.1186/s12918-018-0665-8)
Supplement: Supplementary file 2 — All relevant MATLAB code. A simple Matlab implement of our predictor. (ZIP 19,742 kb) [file 12918_2018_665_MOESM2_ESM.zip › Additional file 2/Read ASA/ASA Property/ASEdb/1A4YA.txt]

 NetSurfP - Protein Surface Accessibility and Secondary Structure Predictions

  

|  |  |  |
| --- | --- | --- |
|  |  | NetSurfP - Protein Surface Accessibility and Secondary Structure Predictions  Technical University of Denmark |

  


---

```
# For publication of results, please cite:
# A generic method for assignment of reliability scores applied to solvent accessibility predictions.
# Bent Petersen, Thomas Nordahl Petersen, Pernille Andersen, Morten Nielsen and Claus Lundegaard
# BMC Structural Biology 2009, 9:51 doi:10.1186/1472-6807-9-51
#
# Column 1: Class assignment - B for buried or E for Exposed - Threshold: 25% exposure, but not based on RSA
# Column 2: Amino acid
# Column 3: Sequence name
# Column 4: Amino acid number
# Column 5: Relative Surface Accessibility - RSA
# Column 6: Absolute Surface Accessibility
# Column 7: Z-fit score for RSA prediction
# Column 8: Probability for Alpha-Helix
# Column 9: Probability for Beta-strand
# Column 10: Probability for Coil
E S  1A4YA                  1    0.898 105.199  -0.268   0.003   0.003   0.994
E L  1A4YA                  2    0.400  73.332   0.537   0.004   0.138   0.858
E D  1A4YA                  3    0.539  77.713   1.445   0.005   0.262   0.733
B I  1A4YA                  4    0.186  34.428   0.190   0.004   0.420   0.576
E Q  1A4YA                  5    0.420  74.941   1.537   0.003   0.718   0.279
E S  1A4YA                  6    0.420  49.201   1.607   0.001   0.959   0.040
B L  1A4YA                  7    0.054   9.924   0.911   0.001   0.959   0.040
E D  1A4YA                  8    0.340  48.936   0.492   0.001   0.900   0.099
B I  1A4YA                  9    0.047   8.621   0.161   0.004   0.616   0.381
E Q  1A4YA                 10    0.372  66.350   0.094   0.004   0.420   0.576
E C  1A4YA                 11    0.600  84.198  -1.078   0.004   0.197   0.799
B E  1A4YA                 12    0.188  32.844   0.270   0.004   0.197   0.799
E E  1A4YA                 13    0.518  90.530   0.334   0.004   0.197   0.799
B L  1A4YA                 14    0.066  12.085  -1.263   0.004   0.085   0.910
E S  1A4YA                 15    0.353  41.407   0.072   0.018   0.019   0.964
E D  1A4YA                 16    0.588  84.731  -1.581   0.502   0.002   0.495
E A  1A4YA                 17    0.635  69.999   0.743   0.782   0.003   0.216
B R  1A4YA                 18    0.183  41.838  -0.001   0.694   0.003   0.303
B W  1A4YA                 19    0.036   8.658   0.209   0.923   0.002   0.076
E A  1A4YA                 20    0.480  52.874   1.520   0.970   0.001   0.030
E E  1A4YA                 21    0.438  76.449   1.223   0.970   0.001   0.030
B L  1A4YA                 22    0.036   6.518  -0.561   0.970   0.001   0.030
B L  1A4YA                 23    0.093  17.102  -0.859   0.970   0.001   0.030
E P  1A4YA                 24    0.622  88.304   1.148   0.923   0.002   0.076
E L  1A4YA                 25    0.298  54.491  -0.007   0.782   0.003   0.216
B L  1A4YA                 26    0.060  10.913  -0.327   0.502   0.002   0.495
E Q  1A4YA                 27    0.569 101.677   1.249   0.502   0.002   0.495
E Q  1A4YA                 28    0.516  92.175   1.837   0.257   0.016   0.727
B C  1A4YA                 29    0.066   9.210   0.885   0.052   0.084   0.864
E Q  1A4YA                 30    0.402  71.833   1.397   0.021   0.279   0.699
E V  1A4YA                 31    0.429  65.953   1.781   0.001   0.900   0.099
B V  1A4YA                 32    0.045   6.963   1.058   0.011   0.918   0.071
E R  1A4YA                 33    0.313  71.631   1.048   0.018   0.846   0.136
B L  1A4YA                 34    0.057  10.400  -0.232   0.021   0.451   0.528
E D  1A4YA                 35    0.408  58.822  -0.433   0.022   0.359   0.619
E D  1A4YA                 36    0.594  85.639  -0.855   0.021   0.279   0.699
B C  1A4YA                 37    0.147  20.653  -0.669   0.004   0.197   0.799
E G  1A4YA                 38    0.476  37.453  -0.000   0.004   0.197   0.799
B L  1A4YA                 39    0.047   8.624  -0.884   0.004   0.085   0.910
E T  1A4YA                 40    0.324  44.980   0.495   0.018   0.019   0.964
E E  1A4YA                 41    0.576 100.575  -1.777   0.694   0.003   0.303
E A  1A4YA                 42    0.665  73.272   0.354   0.694   0.003   0.303
B R  1A4YA                 43    0.166  38.014  -0.682   0.694   0.003   0.303
B C  1A4YA                 44    0.062   8.649  -0.074   0.879   0.010   0.111
E K  1A4YA                 45    0.458  94.272   0.696   0.879   0.010   0.111
E D  1A4YA                 46    0.351  50.507   0.761   0.923   0.002   0.076
B I  1A4YA                 47    0.026   4.810   0.907   0.970   0.001   0.030
B S  1A4YA                 48    0.080   9.329  -0.663   0.970   0.001   0.030
E S  1A4YA                 49    0.553  64.858   0.973   0.970   0.001   0.030
B A  1A4YA                 50    0.111  12.276  -0.886   0.923   0.002   0.076
B L  1A4YA                 51    0.049   9.045  -0.633   0.858   0.002   0.139
E R  1A4YA                 52    0.538 123.179   0.669   0.600   0.003   0.397
E V  1A4YA                 53    0.645  99.152   0.715   0.321   0.003   0.675
B N  1A4YA                 54    0.104  15.196   0.654   0.016   0.005   0.979
E P  1A4YA                 55    0.592  84.062   0.467   0.018   0.019   0.964
E A  1A4YA                 56    0.427  47.044   1.443   0.004   0.085   0.910
B L  1A4YA                 57    0.063  11.554   0.883   0.004   0.138   0.858
E A  1A4YA                 58    0.348  38.306   1.297   0.005   0.262   0.733
E E  1A4YA                 59    0.428  74.824   1.557   0.001   0.900   0.099
B L  1A4YA                 60    0.041   7.470   0.535   0.001   0.959   0.040
E N  1A4YA                 61    0.298  43.554   0.587   0.001   0.900   0.099
B L  1A4YA                 62    0.050   9.118  -0.206   0.004   0.420   0.576
E R  1A4YA                 63    0.352  80.677  -0.195   0.005   0.262   0.733
E S  1A4YA                 64    0.609  71.387  -1.231   0.004   0.138   0.858
B N  1A4YA                 65    0.164  23.936  -0.952   0.004   0.138   0.858
E E  1A4YA                 66    0.534  93.360   0.197   0.004   0.197   0.799
B L  1A4YA                 67    0.064  11.700  -0.979   0.018   0.088   0.893
E G  1A4YA                 68    0.346  27.254   0.029   0.018   0.019   0.964
E D  1A4YA                 69    0.574  82.757  -1.850   0.694   0.003   0.303
E V  1A4YA                 70    0.628  96.462   0.734   0.782   0.003   0.216
B G  1A4YA                 71    0.085   6.682  -0.253   0.858   0.002   0.139
B V  1A4YA                 72    0.040   6.087  -0.374   0.970   0.001   0.030
E H  1A4YA                 73    0.489  88.895   1.254   0.970   0.001   0.030
E C  1A4YA                 74    0.280  39.284   0.813   0.970   0.001   0.030
B V  1A4YA                 75    0.022   3.443   0.785   0.970   0.001   0.030
B L  1A4YA                 76    0.072  13.238  -1.124   0.970   0.001   0.030
E Q  1A4YA                 77    0.556  99.212   1.145   0.970   0.001   0.030
B G  1A4YA                 78    0.122   9.570  -1.715   0.970   0.001   0.030
B L  1A4YA                 79    0.070  12.762  -1.414   0.923   0.002   0.076
E Q  1A4YA                 80    0.544  97.105   0.100   0.858   0.002   0.139
E T  1A4YA                 81    0.611  84.746  -0.328   0.502   0.002   0.495
E P  1A4YA                 82    0.466  66.168  -1.166   0.176   0.004   0.820
E S  1A4YA                 83    0.495  58.014  -1.371   0.018   0.019   0.964
B C  1A4YA                 84    0.283  39.677  -0.112   0.018   0.047   0.935
E K  1A4YA                 85    0.440  90.446   0.996   0.019   0.141   0.840
B I  1A4YA                 86    0.100  18.518   0.577   0.005   0.336   0.660
E Q  1A4YA                 87    0.264  47.186   0.593   0.004   0.420   0.576
E K  1A4YA                 88    0.342  70.391   1.335   0.001   0.900   0.099
B L  1A4YA                 89    0.030   5.438   0.603   0.001   0.959   0.040
B S  1A4YA                 90    0.238  27.929   0.885   0.001   0.900   0.099
B L  1A4YA                 91    0.037   6.830  -0.919   0.004   0.616   0.381
E Q  1A4YA                 92    0.309  55.259  -0.678   0.005   0.336   0.660
E N  1A4YA                 93    0.561  82.130  -1.134   0.005   0.262   0.733
B C  1A4YA                 94    0.110  15.444  -1.440   0.004   0.197   0.799
E C  1A4YA                 95    0.468  65.749   0.064   0.004   0.197   0.799
B L  1A4YA                 96    0.044   8.020  -0.586   0.004   0.085   0.910
E T  1A4YA                 97    0.296  41.014   0.443   0.018   0.019   0.964
E G  1A4YA                 98    0.585  46.047  -1.750   0.694   0.003   0.303
E A  1A4YA                 99    0.643  70.815   0.789   0.782   0.003   0.216
B G  1A4YA                100    0.119   9.373  -0.557   0.782   0.003   0.216
B C  1A4YA                101    0.057   7.975  -0.077   0.938   0.007   0.055
E G  1A4YA                102    0.470  37.013   0.837   0.938   0.007   0.055
E V  1A4YA                103    0.327  50.214   0.778   0.923   0.002   0.076
B L  1A4YA                104    0.029   5.310   0.791   0.970   0.001   0.030
B S  1A4YA                105    0.098  11.462  -0.544   0.970   0.001   0.030
E S  1A4YA                106    0.534  62.585   1.038   0.970   0.001   0.030
B T  1A4YA                107    0.114  15.798  -1.116   0.970   0.001   0.030
B L  1A4YA                108    0.058  10.528  -0.856   0.858   0.002   0.139
E R  1A4YA                109    0.494 113.103   0.249   0.694   0.003   0.303
E T  1A4YA                110    0.656  90.987   0.738   0.406   0.004   0.590
B L  1A4YA                111    0.110  20.196   0.276   0.053   0.005   0.942
E P  1A4YA                112    0.572  81.167   0.396   0.018   0.019   0.964
E T  1A4YA                113    0.416  57.741   1.600   0.004   0.085   0.910
B L  1A4YA                114    0.052   9.540   0.514   0.004   0.197   0.799
E Q  1A4YA                115    0.326  58.277   1.405   0.005   0.336   0.660
E E  1A4YA                116    0.409  71.452   1.487   0.001   0.900   0.099
B L  1A4YA                117    0.039   7.178   0.464   0.001   0.959   0.040
E H  1A4YA                118    0.287  52.242   0.574   0.001   0.900   0.099
B L  1A4YA                119    0.051   9.375  -0.201   0.004   0.420   0.576
E S  1A4YA                120    0.404  47.396  -0.269   0.005   0.262   0.733
E D  1A4YA                121    0.591  85.149  -0.711   0.004   0.138   0.858
B N  1A4YA                122    0.133  19.457  -0.648   0.004   0.138   0.858
E L  1A4YA                123    0.532  97.428   0.375   0.021   0.279   0.699
B L  1A4YA                124    0.078  14.245  -1.412   0.019   0.141   0.840
E G  1A4YA                125    0.355  27.954  -0.319   0.018   0.047   0.935
E D  1A4YA                126    0.514  74.039  -1.952   0.406   0.004   0.590
E A  1A4YA                127    0.633  69.735   0.397   0.694   0.003   0.303
B G  1A4YA                128    0.154  12.088  -0.445   0.694   0.003   0.303
B L  1A4YA                129    0.038   7.013  -0.606   0.923   0.002   0.076
E Q  1A4YA                130    0.457  81.602   0.976   0.923   0.002   0.076
E L  1A4YA                131    0.299  54.784   0.444   0.970   0.001   0.030
B L  1A4YA                132    0.031   5.621   0.132   0.970   0.001   0.030
B C  1A4YA                133    0.058   8.129  -0.840   0.970   0.001   0.030
E E  1A4YA                134    0.510  89.149   1.027   0.970   0.001   0.030
B G  1A4YA                135    0.126   9.948  -1.501   0.970   0.001   0.030
B L  1A4YA                136    0.081  14.813  -1.361   0.923   0.002   0.076
E L  1A4YA                137    0.500  91.532   0.015   0.858   0.002   0.139
E D  1A4YA                138    0.636  91.662   0.115   0.600   0.003   0.397
E P  1A4YA                139    0.395  56.065  -1.100   0.246   0.004   0.750
E Q  1A4YA                140    0.460  82.174  -1.025   0.058   0.017   0.925
E C  1A4YA                141    0.326  45.813  -0.579   0.053   0.043   0.903
E R  1A4YA                142    0.419  95.905   1.147   0.056   0.142   0.802
B L  1A4YA                143    0.076  13.879  -0.267   0.020   0.205   0.775
E E  1A4YA                144    0.312  54.506   0.804   0.022   0.359   0.619
E K  1A4YA                145    0.358  73.579   1.289   0.011   0.918   0.071
B L  1A4YA                146    0.037   6.830   0.280   0.011   0.918   0.071
B Q  1A4YA                147    0.250  44.632   0.699   0.011   0.918   0.071
B L  1A4YA                148    0.042   7.654  -0.737   0.023   0.655   0.322
E E  1A4YA                149    0.293  51.170  -0.742   0.004   0.420   0.576
E Y  1A4YA                150    0.537 114.821  -0.837   0.005   0.336   0.660
B C  1A4YA                151    0.086  12.117  -0.481   0.005   0.262   0.733
E S  1A4YA                152    0.461  54.017  -0.213   0.004   0.197   0.799
B L  1A4YA                153    0.046   8.368  -0.746   0.005   0.045   0.951
E S  1A4YA                154    0.319  37.434  -0.081   0.018   0.019   0.964
E A  1A4YA                155    0.526  57.998  -2.051   0.600   0.003   0.397
E A  1A4YA                156    0.649  71.509   0.498   0.694   0.003   0.303
B S  1A4YA                157    0.123  14.404  -0.735   0.694   0.003   0.303
B C  1A4YA                158    0.055   7.750  -0.126   0.923   0.002   0.076
E E  1A4YA                159    0.475  83.017   0.753   0.923   0.002   0.076
E P  1A4YA                160    0.279  39.562   0.751   0.923   0.002   0.076
B L  1A4YA                161    0.025   4.559   1.057   0.970   0.001   0.030
B A  1A4YA                162    0.072   7.978  -0.926   0.970   0.001   0.030
E S  1A4YA                163    0.538  63.018   1.114   0.970   0.001   0.030
B V  1A4YA                164    0.114  17.553  -1.126   0.970   0.001   0.030
B L  1A4YA                165    0.062  11.389  -0.971   0.923   0.002   0.076
E R  1A4YA                166    0.531 121.599   0.348   0.694   0.003   0.303
E A  1A4YA                167    0.666  73.382   0.683   0.321   0.003   0.675
B K  1A4YA                168    0.111  22.853   0.109   0.016   0.005   0.979
E P  1A4YA                169    0.578  81.990   0.480   0.005   0.015   0.979
E D  1A4YA                170    0.443  63.807   1.774   0.005   0.045   0.951
B F  1A4YA                171    0.045   9.132   0.661   0.004   0.138   0.858
E K  1A4YA                172    0.342  70.370   1.345   0.005   0.262   0.733
E E  1A4YA                173    0.412  72.029   1.566   0.001   0.900   0.099
B L  1A4YA                174    0.034   6.262   0.827   0.001   0.959   0.040
B T  1A4YA                175    0.260  35.993   0.348   0.001   0.900   0.099
B V  1A4YA                176    0.047   7.285  -0.508   0.022   0.552   0.426
B S  1A4YA                177    0.289  33.906  -0.694   0.005   0.336   0.660
E N  1A4YA                178    0.509  74.518  -1.068   0.004   0.197   0.799
B N  1A4YA                179    0.141  20.613  -0.710   0.004   0.138   0.858
E D  1A4YA                180    0.494  71.229   0.104   0.019   0.141   0.840
B I  1A4YA                181    0.066  12.154  -1.354   0.018   0.047   0.935
E N  1A4YA                182    0.326  47.770  -0.128   0.018   0.019   0.964
E E  1A4YA                183    0.498  86.931  -1.495   0.600   0.003   0.397
E A  1A4YA                184    0.630  69.415   0.907   0.782   0.003   0.216
B G  1A4YA                185    0.124   9.767  -0.120   0.858   0.002   0.139
B V  1A4YA                186    0.032   4.903  -0.047   0.923   0.002   0.076
E R  1A4YA                187    0.456 104.538   1.141   0.970   0.001   0.030
E V  1A4YA                188    0.309  47.478   0.554   0.970   0.001   0.030
B L  1A4YA                189    0.031   5.621   0.149   0.970   0.001   0.030
B C  1A4YA                190    0.063   8.831  -1.092   0.970   0.001   0.030
E Q  1A4YA                191    0.571 101.927   0.968   0.970   0.001   0.030
B G  1A4YA                192    0.218  17.117  -1.422   0.923   0.002   0.076
B L  1A4YA                193    0.109  19.976  -1.209   0.782   0.003   0.216
E K  1A4YA                194    0.571 117.496  -0.351   0.600   0.003   0.397
E D  1A4YA                195    0.547  78.779  -0.999   0.246   0.004   0.750
E S  1A4YA                196    0.449  52.599  -1.196   0.109   0.005   0.886
E P  1A4YA                197    0.457  64.792  -1.240   0.058   0.017   0.925
B C  1A4YA                198    0.291  40.856  -0.542   0.053   0.043   0.903
E Q  1A4YA                199    0.402  71.815   0.944   0.052   0.084   0.864
B L  1A4YA                200    0.107  19.647   0.214   0.020   0.205   0.775
E E  1A4YA                201    0.290  50.733   0.811   0.022   0.359   0.619
E A  1A4YA                202    0.347  38.261   1.531   0.011   0.918   0.071
B L  1A4YA                203    0.030   5.401   0.622   0.006   0.962   0.032
E K  1A4YA                204    0.261  53.626   0.879   0.011   0.918   0.071
B L  1A4YA                205    0.036   6.665  -1.316   0.023   0.655   0.322
E E  1A4YA                206    0.325  56.865  -0.533   0.022   0.359   0.619
E S  1A4YA                207    0.560  65.679  -0.635   0.004   0.197   0.799
B C  1A4YA                208    0.095  13.310  -1.210   0.004   0.138   0.858
E G  1A4YA                209    0.446  35.108  -0.017   0.004   0.138   0.858
B V  1A4YA                210    0.052   7.916  -0.933   0.004   0.085   0.910
E T  1A4YA                211    0.303  41.998   0.276   0.005   0.015   0.979
E S  1A4YA                212    0.590  69.183  -1.962   0.502   0.002   0.495
E D  1A4YA                213    0.676  97.383   0.321   0.600   0.003   0.397
B N  1A4YA                214    0.155  22.677  -0.588   0.600   0.003   0.397
B C  1A4YA                215    0.061   8.635  -0.320   0.923   0.002   0.076
E R  1A4YA                216    0.488 111.775   0.754   0.923   0.002   0.076
E D  1A4YA                217    0.323  46.602   0.551   0.923   0.002   0.076
B L  1A4YA                218    0.023   4.211   1.155   0.970   0.001   0.030
B C  1A4YA                219    0.107  14.967  -0.545   0.970   0.001   0.030
E G  1A4YA                220    0.522  41.121   1.017   0.970   0.001   0.030
B I  1A4YA                221    0.104  19.240  -1.169   0.970   0.001   0.030
B V  1A4YA                222    0.064   9.868  -1.082   0.923   0.002   0.076
E A  1A4YA                223    0.540  59.508   0.459   0.782   0.003   0.216
E S  1A4YA                224    0.663  77.762   0.613   0.406   0.004   0.590
B K  1A4YA                225    0.108  22.298  -0.009   0.053   0.005   0.942
E A  1A4YA                226    0.557  61.381   0.484   0.018   0.019   0.964
E S  1A4YA                227    0.435  51.029   1.524   0.018   0.088   0.893
B L  1A4YA                228    0.060  10.968   0.731   0.004   0.138   0.858
E R  1A4YA                229    0.329  75.272   1.289   0.005   0.262   0.733
E E  1A4YA                230    0.410  71.540   1.842   0.001   0.900   0.099
B L  1A4YA                231    0.032   5.951   0.747   0.001   0.900   0.099
E A  1A4YA                232    0.264  29.071   0.809   0.001   0.900   0.099
B L  1A4YA                233    0.047   8.532  -0.164   0.004   0.514   0.481
E G  1A4YA                234    0.312  24.562  -0.779   0.005   0.336   0.660
E S  1A4YA                235    0.575  67.449  -1.044   0.004   0.138   0.858
B N  1A4YA                236    0.143  20.877  -0.478   0.004   0.138   0.858
E K  1A4YA                237    0.514 105.833  -0.007   0.019   0.141   0.840
B L  1A4YA                238    0.075  13.714  -1.816   0.018   0.047   0.935
E G  1A4YA                239    0.370  29.150  -0.405   0.018   0.019   0.964
E D  1A4YA                240    0.535  77.108  -2.225   0.406   0.004   0.590
E V  1A4YA                241    0.638  98.107   0.058   0.694   0.003   0.303
B G  1A4YA                242    0.146  11.482  -0.592   0.782   0.003   0.216
B M  1A4YA                243    0.044   8.744  -0.316   0.923   0.002   0.076
E A  1A4YA                244    0.443  48.786   0.961   0.923   0.002   0.076
E E  1A4YA                245    0.352  61.459   0.473   0.970   0.001   0.030
B L  1A4YA                246    0.042   7.654  -0.182   0.970   0.001   0.030
B C  1A4YA                247    0.059   8.227  -0.726   0.970   0.001   0.030
E P  1A4YA                248    0.553  78.457   0.451   0.970   0.001   0.030
B G  1A4YA                249    0.231  18.172  -1.718   0.923   0.002   0.076
B L  1A4YA                250    0.130  23.766  -1.031   0.858   0.002   0.139
E L  1A4YA                251    0.427  78.257  -0.902   0.694   0.003   0.303
E H  1A4YA                252    0.534  97.098  -1.059   0.321   0.003   0.675
E P  1A4YA                253    0.542  76.853  -0.985   0.109   0.005   0.886
B S  1A4YA                254    0.225  26.323  -1.032   0.018   0.019   0.964
E S  1A4YA                255    0.372  43.645  -0.316   0.018   0.047   0.935
E R  1A4YA                256    0.435  99.661   0.762   0.018   0.088   0.893
B L  1A4YA                257    0.100  18.383   0.362   0.004   0.197   0.799
E R  1A4YA                258    0.284  65.082   0.798   0.004   0.420   0.576
E T  1A4YA                259    0.346  47.990   1.443   0.001   0.900   0.099
B L  1A4YA                260    0.029   5.273   0.657   0.001   0.959   0.040
B W  1A4YA                261    0.244  58.802   0.804   0.001   0.900   0.099
B I  1A4YA                262    0.039   7.252  -0.736   0.023   0.655   0.322
B W  1A4YA                263    0.279  67.051  -0.858   0.005   0.336   0.660
E E  1A4YA                264    0.496  86.634  -0.791   0.004   0.197   0.799
B C  1A4YA                265    0.097  13.563  -1.621   0.004   0.138   0.858
E G  1A4YA                266    0.418  32.881   0.046   0.004   0.138   0.858
B I  1A4YA                267    0.038   7.012  -1.462   0.004   0.085   0.910
E T  1A4YA                268    0.298  41.319   0.749   0.018   0.019   0.964
E A  1A4YA                269    0.622  68.555  -1.797   0.600   0.003   0.397
E K  1A4YA                270    0.651 133.890   0.604   0.694   0.003   0.303
B G  1A4YA                271    0.143  11.278  -0.627   0.694   0.003   0.303
B C  1A4YA                272    0.063   8.845  -0.364   0.858   0.002   0.139
E G  1A4YA                273    0.483  38.044   0.469   0.858   0.002   0.139
E D  1A4YA                274    0.302  43.489   0.709   0.923   0.002   0.076
B L  1A4YA                275    0.027   5.017   0.893   0.970   0.001   0.030
B C  1A4YA                276    0.075  10.530  -0.793   0.970   0.001   0.030
E R  1A4YA                277    0.525 120.156   1.039   0.970   0.001   0.030
B V  1A4YA                278    0.110  16.922  -1.207   0.923   0.002   0.076
B L  1A4YA                279    0.044   8.093  -1.585   0.858   0.002   0.139
E R  1A4YA                280    0.469 107.332   0.153   0.694   0.003   0.303
E A  1A4YA                281    0.650  71.630   0.677   0.406   0.004   0.590
B K  1A4YA                282    0.085  17.423  -0.020   0.053   0.005   0.942
E E  1A4YA                283    0.566  98.863   0.752   0.018   0.019   0.964
E S  1A4YA                284    0.407  47.700   1.651   0.005   0.045   0.951
B L  1A4YA                285    0.045   8.203   0.670   0.004   0.085   0.910
E K  1A4YA                286    0.340  69.917   1.061   0.004   0.197   0.799
E E  1A4YA                287    0.416  72.658   1.525   0.001   0.900   0.099
B L  1A4YA                288    0.035   6.463   0.549   0.001   0.900   0.099
B S  1A4YA                289    0.274  32.160   0.592   0.018   0.846   0.136
B L  1A4YA                290    0.044   7.983  -0.673   0.005   0.336   0.660
E A  1A4YA                291    0.334  36.862  -0.615   0.004   0.138   0.858
E G  1A4YA                292    0.585  46.079  -0.545   0.018   0.088   0.893
B N  1A4YA                293    0.110  16.133  -0.952   0.018   0.088   0.893
E E  1A4YA                294    0.497  86.791   0.112   0.020   0.205   0.775
B L  1A4YA                295    0.063  11.535  -1.680   0.018   0.088   0.893
E G  1A4YA                296    0.421  33.148  -0.043   0.018   0.019   0.964
E D  1A4YA                297    0.543  78.275  -1.873   0.321   0.003   0.675
E E  1A4YA                298    0.619 108.122   0.266   0.600   0.003   0.397
B G  1A4YA                299    0.141  11.105  -0.187   0.694   0.003   0.303
B A  1A4YA                300    0.044   4.893  -0.246   0.923   0.002   0.076
E R  1A4YA                301    0.493 112.874   1.062   0.923   0.002   0.076
E L  1A4YA                302    0.305  55.919   0.531   0.970   0.001   0.030
B L  1A4YA                303    0.030   5.456   0.258   0.970   0.001   0.030
B C  1A4YA                304    0.053   7.385  -0.473   0.970   0.001   0.030
E E  1A4YA                305    0.543  94.949   1.068   0.970   0.001   0.030
B T  1A4YA                306    0.153  21.152  -0.939   0.970   0.001   0.030
B L  1A4YA                307    0.068  12.432  -1.722   0.923   0.002   0.076
E L  1A4YA                308    0.487  89.170  -0.204   0.858   0.002   0.139
E E  1A4YA                309    0.623 108.873  -0.459   0.502   0.002   0.495
E P  1A4YA                310    0.481  68.296  -1.232   0.246   0.004   0.750
E G  1A4YA                311    0.399  31.370  -0.758   0.016   0.005   0.979
E C  1A4YA                312    0.357  50.123  -0.731   0.018   0.019   0.964
E Q  1A4YA                313    0.450  80.388   1.041   0.018   0.088   0.893
B L  1A4YA                314    0.080  14.703  -0.560   0.004   0.197   0.799
E E  1A4YA                315    0.271  47.361   0.732   0.004   0.420   0.576
E S  1A4YA                316    0.357  41.864   1.382   0.001   0.900   0.099
B L  1A4YA                317    0.031   5.639   0.817   0.001   0.959   0.040
E W  1A4YA                318    0.262  62.963   0.682   0.001   0.900   0.099
B V  1A4YA                319    0.036   5.549  -0.858   0.004   0.616   0.381
B K  1A4YA                320    0.258  53.091  -0.756   0.005   0.336   0.660
E S  1A4YA                321    0.523  61.272  -0.668   0.005   0.262   0.733
B C  1A4YA                322    0.088  12.425  -0.891   0.004   0.197   0.799
E S  1A4YA                323    0.462  54.111   0.371   0.005   0.262   0.733
B F  1A4YA                324    0.050   9.955  -0.313   0.004   0.138   0.858
E T  1A4YA                325    0.317  43.926   0.579   0.018   0.019   0.964
E A  1A4YA                326    0.561  61.822  -1.459   0.502   0.002   0.495
E A  1A4YA                327    0.643  70.815   0.666   0.694   0.003   0.303
B C  1A4YA                328    0.109  15.304  -0.274   0.600   0.003   0.397
B C  1A4YA                329    0.044   6.121  -0.216   0.858   0.002   0.139
E S  1A4YA                330    0.473  55.471   1.012   0.923   0.002   0.076
E H  1A4YA                331    0.328  59.645   0.555   0.923   0.002   0.076
B F  1A4YA                332    0.029   5.840   0.866   0.970   0.001   0.030
B S  1A4YA                333    0.062   7.243  -1.075   0.970   0.001   0.030
E S  1A4YA                334    0.541  63.440   1.183   0.970   0.001   0.030
B V  1A4YA                335    0.107  16.369  -1.211   0.923   0.002   0.076
B L  1A4YA                336    0.065  11.883  -1.225   0.923   0.002   0.076
E A  1A4YA                337    0.524  57.723   0.002   0.694   0.003   0.303
E Q  1A4YA                338    0.682 121.877   0.803   0.406   0.004   0.590
B N  1A4YA                339    0.121  17.758   0.153   0.053   0.005   0.942
E R  1A4YA                340    0.554 126.980   0.569   0.018   0.019   0.964
E F  1A4YA                341    0.355  71.309   1.441   0.005   0.045   0.951
B L  1A4YA                342    0.043   7.947   0.501   0.004   0.138   0.858
E L  1A4YA                343    0.323  59.141   1.216   0.005   0.262   0.733
E E  1A4YA                344    0.397  69.391   1.625   0.001   0.900   0.099
B L  1A4YA                345    0.040   7.379   0.424   0.001   0.900   0.099
B Q  1A4YA                346    0.280  49.972   0.404   0.002   0.816   0.182
B I  1A4YA                347    0.050   9.305  -0.113   0.005   0.336   0.660
E S  1A4YA                348    0.290  33.965  -0.787   0.020   0.205   0.775
E N  1A4YA                349    0.555  81.267  -0.460   0.019   0.141   0.840
B N  1A4YA                350    0.107  15.723  -0.898   0.019   0.141   0.840
E R  1A4YA                351    0.495 113.263   0.222   0.019   0.141   0.840
B L  1A4YA                352    0.066  12.011  -1.014   0.018   0.088   0.893
E E  1A4YA                353    0.390  68.133   0.107   0.018   0.019   0.964
E D  1A4YA                354    0.500  72.021  -2.259   0.321   0.003   0.675
E A  1A4YA                355    0.587  64.665   0.429   0.600   0.003   0.397
B G  1A4YA                356    0.113   8.917  -0.320   0.717   0.014   0.269
B V  1A4YA                357    0.043   6.655  -0.018   0.923   0.002   0.076
E R  1A4YA                358    0.409  93.707   1.067   0.923   0.002   0.076
E E  1A4YA                359    0.265  46.383   0.743   0.970   0.001   0.030
B L  1A4YA                360    0.026   4.834   0.670   0.970   0.001   0.030
B C  1A4YA                361    0.056   7.834  -1.198   0.970   0.001   0.030
E Q  1A4YA                362    0.537  95.926   1.266   0.970   0.001   0.030
B G  1A4YA                363    0.156  12.261  -1.262   0.970   0.001   0.030
B L  1A4YA                364    0.071  12.982  -0.875   0.923   0.002   0.076
E G  1A4YA                365    0.538  42.309   0.249   0.782   0.003   0.216
E Q  1A4YA                366    0.649 115.876   0.356   0.502   0.002   0.495
E P  1A4YA                367    0.370  52.446  -0.626   0.321   0.003   0.675
B G  1A4YA                368    0.312  24.515  -0.590   0.058   0.017   0.925
E S  1A4YA                369    0.413  48.392  -0.399   0.053   0.043   0.903
E V  1A4YA                370    0.407  62.556   1.369   0.052   0.084   0.864
B L  1A4YA                371    0.075  13.824   0.210   0.020   0.205   0.775
E R  1A4YA                372    0.333  76.143   1.221   0.004   0.420   0.576
E V  1A4YA                373    0.375  57.699   1.595   0.001   0.900   0.099
B L  1A4YA                374    0.035   6.354   0.581   0.001   0.959   0.040
B W  1A4YA                375    0.256  61.520   0.587   0.001   0.900   0.099
B L  1A4YA                376    0.045   8.258  -0.725   0.004   0.514   0.481
E A  1A4YA                377    0.304  33.479  -0.685   0.005   0.336   0.660
E D  1A4YA                378    0.559  80.595  -0.639   0.004   0.197   0.799
B C  1A4YA                379    0.098  13.787  -1.565   0.004   0.138   0.858
E D  1A4YA                380    0.443  63.836  -0.039   0.004   0.197   0.799
B V  1A4YA                381    0.046   7.009  -0.653   0.004   0.085   0.910
E S  1A4YA                382    0.276  32.300   0.512   0.018   0.019   0.964
E D  1A4YA                383    0.547  78.794  -1.700   0.600   0.003   0.397
E S  1A4YA                384    0.638  74.820   0.544   0.694   0.003   0.303
B S  1A4YA                385    0.113  13.244  -0.842   0.694   0.003   0.303
B C  1A4YA                386    0.054   7.652  -0.508   0.858   0.002   0.139
E S  1A4YA                387    0.399  46.739   0.368   0.858   0.002   0.139
E S  1A4YA                388    0.299  35.055   0.684   0.923   0.002   0.076
B L  1A4YA                389    0.024   4.339   1.141   0.970   0.001   0.030
B A  1A4YA                390    0.064   7.064  -0.688   0.970   0.001   0.030
E A  1A4YA                391    0.528  58.186   1.175   0.970   0.001   0.030
B T  1A4YA                392    0.109  15.077  -0.881   0.970   0.001   0.030
B L  1A4YA                393    0.052   9.485  -0.661   0.923   0.002   0.076
E L  1A4YA                394    0.509  93.106   0.635   0.782   0.003   0.216
E A  1A4YA                395    0.671  73.999   0.811   0.502   0.002   0.495
B N  1A4YA                396    0.121  17.700   0.546   0.053   0.005   0.942
E H  1A4YA                397    0.592 107.630   0.764   0.018   0.019   0.964
E S  1A4YA                398    0.443  51.896   1.806   0.005   0.045   0.951
B L  1A4YA                399    0.044   7.965   0.630   0.004   0.085   0.910
E R  1A4YA                400    0.356  81.593   1.532   0.004   0.197   0.799
E E  1A4YA                401    0.423  73.881   1.683   0.001   0.900   0.099
B L  1A4YA                402    0.037   6.848   0.676   0.001   0.959   0.040
B D  1A4YA                403    0.262  37.783   0.585   0.001   0.900   0.099
B L  1A4YA                404    0.040   7.361  -0.172   0.004   0.420   0.576
E S  1A4YA                405    0.329  38.594  -0.628   0.005   0.262   0.733
E N  1A4YA                406    0.563  82.394  -0.524   0.004   0.085   0.910
B N  1A4YA                407    0.112  16.411  -0.828   0.004   0.085   0.910
E C  1A4YA                408    0.511  71.772   0.581   0.004   0.138   0.858
B L  1A4YA                409    0.050   9.118  -0.831   0.005   0.045   0.951
E G  1A4YA                410    0.365  28.733   0.227   0.018   0.019   0.964
E D  1A4YA                411    0.548  78.895  -1.848   0.694   0.003   0.303
E A  1A4YA                412    0.611  67.343   0.854   0.858   0.002   0.139
B G  1A4YA                413    0.088   6.894  -0.040   0.858   0.002   0.139
B I  1A4YA                414    0.048   8.843   0.125   0.970   0.001   0.030
E L  1A4YA                415    0.473  86.606   1.314   0.970   0.001   0.030
E Q  1A4YA                416    0.285  50.937   1.232   0.970   0.001   0.030
B L  1A4YA                417    0.022   4.083   0.973   0.970   0.001   0.030
B V  1A4YA                418    0.100  15.309  -1.078   0.970   0.001   0.030
E E  1A4YA                419    0.554  96.784   1.452   0.970   0.001   0.030
B S  1A4YA                420    0.099  11.615  -1.048   0.970   0.001   0.030
B V  1A4YA                421    0.074  11.389  -1.347   0.923   0.002   0.076
E R  1A4YA                422    0.615 140.927   0.655   0.782   0.003   0.216
E Q  1A4YA                423    0.646 115.447   0.722   0.600   0.003   0.397
E P  1A4YA                424    0.281  39.846  -0.680   0.321   0.003   0.675
E G  1A4YA                425    0.467  36.784  -0.500   0.115   0.016   0.868
E C  1A4YA                426    0.457  64.107   0.014   0.268   0.043   0.689
E L  1A4YA                427    0.352  64.396   1.386   0.184   0.043   0.773
B L  1A4YA                428    0.061  11.114   0.330   0.052   0.084   0.864
E E  1A4YA                429    0.407  71.068   1.058   0.021   0.279   0.699
E Q  1A4YA                430    0.365  65.260   1.923   0.018   0.846   0.136
B L  1A4YA                431    0.033   6.024   0.722   0.011   0.918   0.071
E V  1A4YA                432    0.287  44.081   0.788   0.018   0.846   0.136
B L  1A4YA                433    0.048   8.844  -0.323   0.021   0.451   0.528
E Y  1A4YA                434    0.416  89.006  -0.168   0.020   0.205   0.775
E D  1A4YA                435    0.617  88.939  -1.025   0.004   0.138   0.858
B I  1A4YA                436    0.138  25.622  -0.539   0.004   0.138   0.858
E Y  1A4YA                437    0.495 105.739   0.137   0.004   0.197   0.799
B W  1A4YA                438    0.064  15.368  -0.568   0.004   0.085   0.910
E S  1A4YA                439    0.349  40.950   0.427   0.018   0.019   0.964
E E  1A4YA                440    0.601 105.012  -1.199   0.923   0.002   0.076
E E  1A4YA                441    0.560  97.780   1.031   0.923   0.002   0.076
B M  1A4YA                442    0.058  11.646   0.438   0.923   0.002   0.076
B E  1A4YA                443    0.091  15.863   0.770   0.970   0.001   0.030
E D  1A4YA                444    0.541  77.958   1.349   0.970   0.001   0.030
B R  1A4YA                445    0.224  51.296   1.245   0.970   0.001   0.030
B L  1A4YA                446    0.024   4.321   0.906   0.970   0.001   0.030
E Q  1A4YA                447    0.249  44.525   0.480   0.988   0.000   0.012
E A  1A4YA                448    0.525  57.910   1.602   0.970   0.001   0.030
B L  1A4YA                449    0.070  12.744  -0.820   0.970   0.001   0.030
B E  1A4YA                450    0.182  31.813  -0.348   0.923   0.002   0.076
E K  1A4YA                451    0.659 135.597   0.914   0.858   0.002   0.139
E D  1A4YA                452    0.634  91.431   1.011   0.600   0.003   0.397
B K  1A4YA                453    0.164  33.714   0.709   0.109   0.005   0.886
E P  1A4YA                454    0.608  86.275   0.579   0.016   0.005   0.979
E S  1A4YA                455    0.525  61.495   1.750   0.018   0.047   0.935
B L  1A4YA                456    0.081  14.849   0.146   0.004   0.085   0.910
E R  1A4YA                457    0.520 119.149   1.578   0.021   0.279   0.699
E V  1A4YA                458    0.498  76.619   0.832   0.021   0.279   0.699
B I  1A4YA                459    0.207  38.350   0.755   0.018   0.088   0.893
E S  1A4YA                460    0.739  86.576  -1.529   0.003   0.003   0.994

Explain the output.  Go back.
```

---
